# Assessment of nodal target definition and dosimetry using three different techniques: implications for re-defining the optimal pelvic field in endometrial cancer

**DOI:** 10.1186/1748-717X-5-59

**Published:** 2010-06-27

**Authors:** Susan Guo, Ronald D Ennis, Stephen Bhatia, Frieda Trichter, Benjamin Bashist, Jinesh Shah, Manjeet Chadha

**Affiliations:** 1 Department of Radiation Oncology, St. Luke's-Roosevelt Hospital Center, Continuum Health Partners, New York NY, USA; 2 Columbia University College of Physicians and Surgeons, New York, NY, USA; 3 Department of Radiology, St. Luke's-Roosevelt Hospital Center, Continuum Health Partners, New York, NY, USA; 4 Department Radiation Oncology, Columbia University College of Physicians and Surgeons, New York, NY, USA; 5 Department of Radiation Oncology, Beth Israel Medical Center, Continuum Health Partners, New York, NY, USA

## Abstract

**Purposes:**

1. To determine the optimal pelvic nodal clinical target volume for post-operative treatment of endometrial cancer. 2. To compare the DVH of different treatment planning techniques applied to this new CTV and the surrounding tissues.

**Methods and Materials:**

Based on the literature, we selected a methodology to delineate nodal target volume to define a NEW-CTV and NEW-PTV. Conventional 2D fields, 3D fields based on anatomic guidelines per RTOG 0418, 3D fields based on our guidelines, and IMRT based on our guidelines were assessed for coverage of NEW-CTV, NEW-PTV, and surrounding structures. CT scans of 10 patients with gynecologic malignancies after TAH/BSO were used. DVHs were compared.

**Results:**

For NEW-PTV, mean V45Gy were 50% and 69% for 2D and RTOG 0418-3DCRT vs. 98% and 97% for NEW-3DCRT and NEW-IMRT (p < 0.0009). Mean V45Gy small bowel were 24% and 20% for 2D and RTOG 0418-3DCRT, increased to 32% with NEW-3DCRT, and decreased to 14% with IMRT (p = 0.005, 0.138, 0.002). Mean V45Gy rectum were 26%, 35%, and 52% for 2D, RTOG 0418-3DCRT, and NEW-3DCRT, and decreased to 26% with NEW-IMRT (p < 0.05). Mean V45Gy bladder were 83%, 51%, and 73% for 2D, RTOG 0418-3DCRT, and NEW-3DCRT, and decreased to 30% with NEW-IMRT (p < 0.002).

**Conclusions:**

Conventional 2D and RTOG 0418-based 3DCRT plans cover only a fraction of our comprehensive PTV. A 3DCRT plan covers this PTV with high doses to normal tissues, whereas IMRT covers the PTV while delivering lower normal tissue doses. Re-consideration of what *specifically *the pelvic target encompasses is warranted.

## Background

Whole pelvic radiation therapy (WPRT) is commonly used in the post-operative treatment of endometrial malignancies as adjuvant therapy targeting the pelvic lymph nodes, in addition to the vaginal apex. Conventional pelvic fields reference anatomical structures of the bones to establish the pelvic radiation therapy fields. Data from studies including lymphangiograms, intraoperative measurements, or placed surgical clips have found that radiation fields determined by bony landmarks alone results in suboptimal coverage of the nodal areas [1-4]. With routine use of CT simulation, contouring vessels on the CT image is used as surrogate for lymph node localization. This method provides more complete, precise, and individualized field delineation compared to that achieved when using conventional pelvic fields[5]. Nevertheless, attempting to do this raises several issues that were heretofore not addressed.

Specifically, it is undefined exactly which nodal regions are the targets for post-operative treatment of endometrial cancer. We undertook a review of the literature for studies that rigorously addressed this issue. We then used these data to define our pelvic nodal CTV and PTV. We then applied conventional, 3D conformal, and IMRT techniques to compare the ability of these techniques to cover this nodal CTV and to determine the normal tissue dosimetric consequences.

## Methods

### Definition of target structures and subsequent planning

We comprehensively reviewed the literature for articles that identified the locations of the pelvic lymph nodes rather than assuming a location. Once the papers were identified, the information from these papers was used to define new CTVs and PTVs. We then planned treatments and determined the dosimetric coverage of these new CTVs and PTVs and the surrounding normal structures. We planned using four different methods: 1)Conventional, 2) 3D-conformal as defined by RTOG 0418, 3) 3D-conformal based on these newly defined CTVs and PTVs, and 4) IMRT based on these new CTVs and PTVs. The treatment planning system used was Pinnacle^3 ^Version 6.2b. For IMRT, the dose calculation algorithm is the Collapsed Cone Convolution Superposition dose algorithm. 18 MV photons were used.

### Patient selection

The CT scans of 10 consecutive patients from our institution with gynecologic malignancies who have undergone TAH/BSO with or without pelvic and para-aortic lymph node dissections were obtained under an IRB-approved protocol.

### Statistical Analysis

The paired-samples test was used to compare dose received to the target volumes and normal structures among the different plans.

## Results

### Literature review

We found three papers that defined the location of the lymph nodes from the vessels. In the paper by Portaluri et al., an extensive analysis of nodal location was performed[6]. However, the patients analyzed in this study had advanced disease and a variety of malignancies. Thus, these data may not be an ideal source for locating clinically uninvolved pelvic nodes in patients with gynecologic malignancies. Taylor et al., however, specifically located the unenlarged nodes in gynecologic patients using ultrasmall superparamagnetic iron oxide particle infusion prior to MRI[7]. This paper, however, did not address the cranial and caudal borders of the relevant lymph nodes. The paper by Shih et al. also addressed the locations of the pelvic nodes using the iron oxide infusion with pelvic MRI in a series of prostate cancer patients[8]. Besides addressing the radial distance from the pelvic vessels this analysis also assessed the cranial and caudal extent. We combined these two sources, with some minor modifications, in defining our lymph node CTVs as is detailed below. Of note, the Taylor group has recently validated their recommendations on another cohort, further strengthening their recommendations[9]. Another recently published paper using this method has similar recommendations[10].The different definitions of the pelvic nodal CTVs are summarized in Table 1.

**Table 1:** Various Guidelines for Pelvic Node CTV Drawing

	Common Iliac	External Iliac	Internal Iliac	Obturator
Portaluri*	Cranial: Aortic bifurcation	Cranial: Common iliac bifurcation (L5-S1)	Cranial: Common iliac bifurcation (L5-S1)	Cranial: Cranial sections of obturator muscle
	Caudal: Common iliac bifurcation	Caudal: Femoral ring (disappearance of lateral muscles of abdominal wall, artery becomes lateral)	Caudal: Cranial sections of coccygeal muscle	Caudal: Superior margin inferior branch of pubic bone
	Anterior: Mesocolon	Anterior: Fat of small bowel, deferent duct or round ligament	Anterior: Bladder, uterus	Anterior: External iliac vein
	Lateral: Psoas muscles	Lateral:	Lateral:	Lateral:
	Posterior: sacrum	- Cranial: Psoas, int iliac vein, iliac bone, sacroiliac joint	- Cranial: Psoas muscle, int iliac vein, iliac bone, sacroiliac joint	- Cranial: Acetabulum
		- Caudal : Piriformis m., internal obturatorius m.	- Caudal : Piriformis m., int obturatorius m.	- Caudal: Internal obturator muscle
		Posterior:	Posterior:	Posterior: Internal obturator muscle
		- Cranial: Ext iliac v	- Cranial: Sacral wing	Medial: Bladder
		- Caudal: Pubic bone (superior branch)	- Caudal: Piriform muscle	
		Medial: Mesocolon, uterus, bladder	Medial: Mesocolon, uterus, bladder	

**Taylor†**	7 mm around common iliac vessels, extending posterior and lateral borders to psoas and vertebral body	7 mm around ext iliac vessels, extending anterior border by additional 10 mm anterolaterally along ilopsoas muscle to include lateral external iliac nodes	7-mm margin around int iliac vessels, extending lateral borders to pelvic sidewall	18-mm wide strip along pelvic sidewall joining external and internal iliac regions

**Shih††**	2.0 cm expansion around the distal 2.5 cm of common iliac vessels superior to bifurcation	2.0 cm expansion around ext iliac vessels for 9 cm from common iliac bifurcation	2.0 cm expansion around int iliac vessels for 8.5 cm extending from common iliac bifurcation	Not specified

**RTOG 0418||**	7 mm around common iliac vessels, with superior border at 7 mm below L4-L5 interspace	7 mm around ext iliac vessels, terminating at level of femoral head	7 mm around int iliac vessels	Not specified

* Portaluri M BS, Perez C, et al. A three-dimensional definition of nodal spaces on the basis of CT images showing enlarged nodes for pelvic radiotherapy. *Int J Radiat Oncol Biol Phys *2005;63:1101-1107.

† Taylor A RA, Reznek RH, et al. Mapping pelvic lymph nodes: Guidelines for delineation in intensity-modulated radiotherapy. *Int J Radiat Oncol Biol Phys *2005;63:1604-1612.

†† Shih HA HM, Zietman AL, et al. Mapping of nodal disease in locally advanced prostate cancer: Rethinking the clinical target volume for pelvic nodal irradiation based on vascular rather than bony anatomy. *Int J Radiat Oncol Biol Phys *2005;63:1262-1269.

|| Jhingren A, Winter K, Portelance L. A phase II study of intensity modulated radiation therapy (IMRT) to the pelvic for post-operative patients with endometrial carcinoma (RTOG 0418). *Int J Radiat Oncol Biol Phys *2008; 72:S16-S17

### Definition of Clinical Target Volumes

#### Common iliac nodes

**Figure 1:**
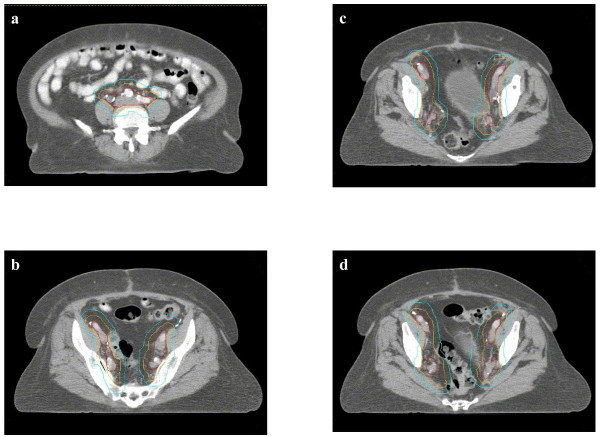
**Defining the NEW-CTV**. a. Common iliac nodes: 7 mm margin around common iliac vessels, extending posterolaterally to the psoas muscle and vertebral body b. Internal iliac nodes: 5 mm margin around internal iliac vessels, extending laterally to pelvic sidewall c. External iliac nodes: 7 mm around external iliac vessels, extending anteriorly along the iliopsoas muscle by additional 10 mm d. Obturator nodes: 15 mm margin joining corresponding medial and lateral borders of internal and external iliac contours. Red: Vessels; Orange: CTV expansion; Cyan: PTV expansion

7-mm margin uniformly surrounding the common iliac vessels, extending posterolaterally to the psoas muscle and vertebral body (Figure 1a). The superior extent begins at the distal 2.5 cm of these vessels.

#### Internal iliac nodes

5-mm margin uniformly surrounding the internal iliac vessels, extending laterally to the pelvic sidewall (Figure 1b). Although the Taylor group recommended a 7-mm margin around the vessels, their data showed that a 5-mm margin covered 93.8% of lymph nodes (vs. 98.6% with 7-mm), so we used the smaller margin in our study[7]. The inferior extent reaches 8 cm below the bifurcation of the common iliac into the internal iliac vessels

#### External iliac nodes

7-mm margin uniformly around external iliac vessels, extending anteriorly along the ilopsoas muscle by another 10 mm (a total of 17 mm from the vessel) (Figure 1c). The inferior extent reaches 8 cm below the bifurcation of the common iliac into the external iliac vessels.

#### Obturator nodes

15-mm margin joining corresponding medial and lateral borders of internal and external iliac contours, creating a single volume on each side of the pelvis (Figure 1d). Although the Taylor group recommended an 18-mm margin joining the internal and external iliac contours contours, their data showed that a 15-mm margin covered 95% of lymph nodes (vs. 99% with 18-mm), so we used the smaller margin in our study[7].

The upper 3 cm of vagina (which was identified by the installation of contrast) was contoured as CTV. The combined volumes for all nodal regions specified above and the upper vagina is termed NEW-CTV. All CTV expansions above were modified to exclude bowel and bone.

### Planning Target Volume

The planning target volume based on the NEW-CTV is termed NEW-PTV and is defined as 7 mm around the NEW-CTV and 10 mm around the upper 3 cm of vagina.

### Normal Structure Volumes

The rectum was delineated from the anal margin to the sigmoid flexure, and the entire bladder was contoured. The small and large bowels were contoured on all slices until 2 cm superior to the CTV. Small bowel and large bowel were contoured separately. Oral contrast was used for delineation of small bowel.

### Plans

**Figure 2:**
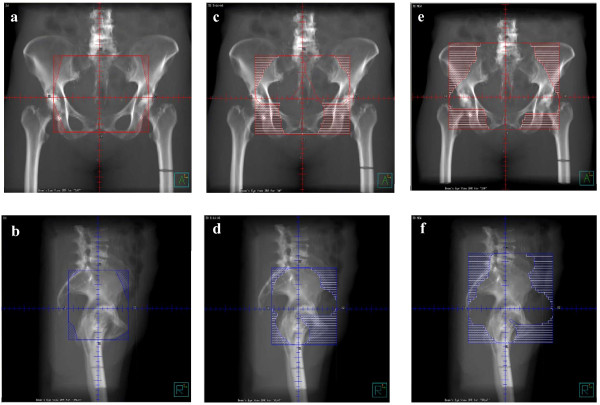
**Comparison of 2D, RTOG 0418-3DCRT, and NEW-3DCRT plans for one patient**. AP and lateral views of 2D plan **(a,b) **AP and lateral views of RTOG 0418-3DCRT plan **(c,d) **AP and lateral views of NEW-3DCRT plan **(e,f)**

Four types of radiation plans were generated for each patient:

1) 2D - 2D fields as done in Finlay et al. (with the addition of corner blocks) i.e. four fields with the borders of the fields extending from the L5-S1 interspace to the bottom of the obturator foramen, and the front of the pubic symphysis to the S2-S3 interspace with standard blocking (Figure 2a and 2b).

2) RTOG 0418-3DCRT - the nodes at risk and upper 3 cm of vagina were contoured as per RTOG 0418 guidelines[11]. 7 mm was added to the vessels and 10 mm were added to the vagina to create the CTV. Four fields were used with the block edges 5 mm beyond the CTV to account for the penumbra. The superior extent of the CTV was 7 mm below L5-S1. The inferior extent of the CTV was limited so that the CTV ended at the top of the femoral heads (Figure 2c and 2d).

3) NEW-3DCRT - Four field 3D plan with the CTV as the newly defined nodal volumes based on literature review above (Figure 2e and 2f)

4) NEW-IMRT - Seven field IMRT plan based on same volumes as in 3. The IMRT parameters were as follows: In addition to the PTV, two control structures were created. The first control structure is a 2 cm "ring" (termed RING) which is created around the PTV. The second control structure is an outer ring which encompasses all tissue beyond the RING. (This structure is called OUTER-RING,PTV.) The PTV had UNIFORM dose objective of 4500 cGy with 100% weighting. The RING had MAXIMUM DOSE objective of 4450 cGy to 1% of volume with 100% weighting. The OUTER-RING,PTV had a MAXIMUM DOSE objective of 2250 cGy to 1% of volume with 100% weighting.

### Dosimetry (Table 2)

**Table 2:** Mean V45Gy Coverage of Target and Normal Structures among Different Plans

	2D	RTOG 0418-3DCRT	NEW-3DCRT	NEW-IMRT
**NEW-PTV**	50%	69%	98%	97%
	(p < 0.0009)	(p < 0.0009)	(p = NS)	
**Small Bowel**	24%	20%	32%	14%
	(p = 0.019)	(p < 0.0009)	(p < 0.0009)	
**Rectum**	26%	35%	52%	26%
	(p = NS)	(p = 0.002)	(p = 0.016)	
**Bladder**	83%	51%	73%	30%
	(p = NS)	(p = NS)	(p < 0.0009)	

Definitions: 2D -four fields with borders extending from the L5-S1 interspace to the bottom of the obturator foramen, and the front of the pubic symphysis to the S2-S3 interspace with standard blocking. RTOG 0418-3DCRT - the nodes at risk and upper 3 cm of vagina contoured as per RTOG 0418 guidelines, with 7 mm added to the vessels and 10 mm to the vagina to create the CTV. Four fields were used with the block edges 5 mm beyond the CTV to account for the penumbra. Superior extent of the CTV was 7 mm below L5-S1. Inferior extent of CTV was limited so that the CTV ended at the top of the femoral heads. NEW-3DCRT - Four field 3D plan with the CTV based on our newly defined nodal volumes. NEW-IMRT - Seven field IMRT plan with the CTV based our newly defined nodal volumes.

All of the V45Gy reported were mean values from the 10 patients analyzed. P values reported were from comparison against the NEW-IMRT plan.

Both the 2D and RTOG 0418-3DCRT plans covered only a fraction of the NEW-PTV. The NEW-PTV mean V45Gy were 50% and 69% for 2D and RTOG 0418-3DCRT (p < 0.0009 both, compared to NEW-IMRT) compared to 98% and 97% for the NEW-3D and the NEW-IMRT plans, respectively.

Part of the reason for this was incomplete coverage of the distal 2.5 cm of the common iliac nodes which should be included in the CTV based on Shih et al[8]. The superior border of the RTOG 0418-3DCRT plan was defined as L4-L5. With this border, 5/10 patients had 100% coverage of the distal 2.5 cm of common iliac vessels, 1/10 patients had 0% coverage, and 4/10 patients had partial coverage (36%, 48%, 60%, and 84%). When this border was placed at L5-S1 as is sometimes done in clinical practice, 9/10 patients had 0% coverage and 1/10 patients had partial coverage (25%).

Mean V45Gy for small bowel was 24% for 2D and 20% for RTOG 0418-3DCRT. To cover NEW-PTV with 3D planning (i.e. NEW-3DCRT), the mean small bowel V45Gy increased to 32% (p = 0.006 vs. 2D and < 0.0009 vs. RTOG 0418-3DCRT). However, NEW-IMRT decreased this to 14% while still covering the NEW-PTV (p = 0.005, p = 0.138 and p = 0.002 for 2D, RTOG 0418-3DCRT and NEW-3DCRT, respectively, vs. NEW-IMRT).

For the rectum, mean 2D V45Gy was 26%. RTOG 0418-3DCRT and NEW-3DCRT increased mean rectal V45Gy to 35% and 52%, respectively (both p = NS compared to 2D). NEW-IMRT was able to decrease mean rectal V45Gy back to 26% (p = 0.002 and 0.016 compared with RTOG 0418-3DCRT and NEW-3DCRT, respectively). For the bladder, mean V45Gy for 2D and RTOG 0418-3DCRT were 83% and 51% (p = 0.049). The NEW-3DCRT mean bladder V45Gy is 73% (p = NS vs. 2D and RTOG-3DCRT), but NEW-IMRT was able to decrease this to 30% (all p < 0.002).

## Discussion

Our study had three goals. Our first goal was to comprehensively review the literature and define pelvic nodal region clinical target volumes and planning target volumes. In our opinion, the ultrasmall superparamagnetic iron oxide particle infusion prior to MRI method, as used by Taylor et al. and Shih et al., is superior to all others for determining the location of these clinically uninvolved nodes[7,8]. However, developing a comprehensive definition of the target volumes using this technique in all dimensions (cranial, caudal, and radial) has not been done. Specifically, Taylor et al. did not determine the cranial and caudal extent of the nodes at risk which in our view is an important unaddressed issue. The results of Shih et al. provide this information. Conversely, Shih at al. do not provide circumferential volumes needed to create CTVs with the same level of detail as provided by Taylor et al. Therefore, we recommend a combination of the two as the optimal method to create accurate CTVs and PTVs.

Our second goal was to test the dosimetric implications of adopting these detailed recommendations for target definition of different nodal CTV groups proposed by Taylor et al and Shih et al. Our analysis shows that complete coverage of the nodes at risk is not achieved with current treatment techniques. Furthermore, any attempt to do this with 2D or 3D-CRT techniques would result in significantly higher doses to the rectum and small bowel which would likely increase the complication rates. However, we have shown that using IMRT, the nodes at risk can be completely covered while actually decreasing the doses to the surrounding normal tissues compared with current standards of care. This would be expected to actually decrease the complications of pelvic radiotherapy compared with standard treatment.

Our findings are consistent with other dosimetric studies that compared IMRT to standard 2D and 3DCRT plans, although these studies defined the CTV differently[12-14]. Portelance et al reported a 48-67% reduction in volume of small bowel irradiated to more than 45 Gy with IMRT vs. standard 2D plan[14]. Roeske et al reported a 50% reduction in volume of small bowel irradiated to more than 45 Gy with IMRT vs. standard 3DCRT[12]. Our results showed a 42% reduction in volume of small bowel irradiated to 45 Gy with IMRT (designed to comprehensively irradiate the pelvis as proposed in this study) compared with a standard 2D plan, and a 30% reduction in volume of small bowel irradiated to 45 Gy with IMRT compared with a 3DCRT plan that defined the CTV based on the small volumes recommended in RTOG-0418.

Our third goal was to assess the reasons for these dosimetric differences and to consider the clinical relevance of our observations. The differences in CTV coverage between the different methods is due in large part to three issues: 1) the cranial extent of the target common iliac nodes 2) the caudal extent of the target external iliac nodes and 3) the need (and, if needed, the extent) to treat the internal iliac nodes. The external iliac lymph nodes are an acknowledged target, but the caudal extent of coverage of a typical radiotherapy port does not cover the caudal extent of this nodal chain. Similarly, the common iliac nodes are often a stated target and yet a variable cranial extent of these nodes is encompassed in a typical pelvic field that sets the superior border at L4-L5 or L5-S1, as is commonly done. Similarly, what portion, if any, of the internal iliac lymph nodes should be included in the defined target is not well-defined.

While our analysis provides a rational method for determining the target nodal structures, it suggests that a larger treatment field is required to encompass all the intended nodal stations that should be included as described in the historic literature. It should be acknowledged that excellent outcome data using conventional pelvic fields are reported in the published literature. For example, the PORTEC trial showed five-year locoregional recurrence rates of only 4% in those randomized to post-operative pelvic radiotherapy[15]. Postoperative radiotherapy in this trial was delivered using a conventional technique with a superior border at L5-S1 via a three-field or a four-field box. In addition, the recent MRC/NCIC trial of patients with intermediate-risk or high-risk early-stage endometrial cancer randomized to surgery with and without pelvic radiation therapy showed 5-year cumulate incidence of isolated vaginal or pelvic recurrence was only 2.9% in those randomized to post-operative radiotherapy[16]. The actuarial total pelvic failure rate is not given. However, the crude number of isolated pelvic recurrences is 13 and the total number of pelvic recurrences is 20; so the actuarial total pelvic recurrence rate in those who received post-operative conventional pelvic radiotherapy is about 4.5%. No information was reported regarding the treatment fields in their analysis but it is likely that conventional techniques were used.

These low recurrence rates suggest that it may not be necessary treat as extensive a volume as we suggest here. Specifically, since the outcome is so good even though the common and internal iliac nodes are partially and variably covered in conventional treatment, a reasonable conclusion would be that treatment of these nodal regions is not needed in the typical node negative, high risk, endometrial cancer patient receiving post-operative pelvic radiotherapy. Further studies on patterns of failure would be valuable to address this question. In the absence of data from a pattern of failure analysis suggesting otherwise, a reasonable approach would be to exclude the common and internal iliac nodal regions from the target structures. This might also allow treatment of smaller treatment volumes and thereby potentially decrease complications while maintaining the excellent results of conventional treatment. A prospective evaluation of this approach is recommended to determine the efficacy and morbidity of this approach and we hope to undertake such a study.

## Conclusions

The nodal tissue at risk can be defined based on a combination of data from Taylor et al and Shih et al[7,8]. Treating these tissues in their entirety would result in significant increases in dose to normal tissues if conventional or 3D techniques are used. The use of IMRT would further decrease the dose to acceptable thresholds. In light of the very good results with conventional treatment which does not treat all the stated nodal targets, further work is needed to define the ideal target lymph node stations when the primary tumor has high risk factors but the nodes are pathologically negative.

## Competing interests

The authors declare that they have no competing interests.

## Authors' contributions

SG contoured the volumes for each patient, performed the statistical analysis, and drafted the manuscript. RDE conceived, designed, and coordinated the study, guided the statistical analysis, and edited the manuscript. SB and FB participated in the treatment planning for all patients. BB assisted in delineating the contours. JS participated in earlier studies that led to the development of this study and edited the manuscript. MC edited the manuscript. All authors read and approved the final manuscript.
